# Association of type 2 diabetes with liver cirrhosis: a nationwide cohort study

**DOI:** 10.18632/oncotarget.18466

**Published:** 2017-06-13

**Authors:** Ping-Hsin Hsieh, Jing-Yang Huang, Oswald Ndi Nfor, Chia-Chi Lung, Chien-Chang Ho, Yung-Po Liaw

**Affiliations:** ^1^ Department of Internal Medicine, Division of Gastroenterology and Hepatology, Chi-Mei Medical Center, Tainan, Taiwan; ^2^ Department of Public Health and Institute of Public Health, Chung Shan Medical University, Taichung, Taiwan; ^3^ Department of Family and Community Medicine, Chung Shan Medical University Hospital, Taichung, Taiwan; ^4^ Department of Physical Education, Fu-Jen Catholic University, New Taipei, Taiwan

**Keywords:** alcohol, cirrhosis, type 2 diabetes, public health, prevention

## Abstract

**Background:**

The link between the subcategories of liver cirrhosis and type 2 diabetes is not well known. We investigated the association of type 2 diabetes mellitus with alcoholic cirrhosis and cirrhosis without alcohol.

**Methods:**

This nationwide cohort study used the Taiwan National Health Insurance Research Database. Cirrhotic individuals and their matched controls were identified from 2001-2008. In all, 9 313 cirrhotic patients aged 20 years or older were matched by age, sex, and index date with the non-cirrhotic individuals (*n* = 37 252). Cirrhosis was categorized into alcoholic cirrhosis and cirrhosis without alcohol. Type 2 diabetes mellitus was identified from January 2001- December 2011.

**Results:**

The incidence densities (per 1 000 person-months) of type 2 diabetes were as follows: 1.14 (95% CI: 1.09-1.20) in the non-cirrhotic group, 1.88 (CI 1.76-2.01) in patients with cirrhosis, 1.62 (CI 1.48-1.78) in patients with cirrhosis without alcohol, and 2.92 (CI 2.64-3.23) in patients with alcoholic cirrhosis. The adjusted hazards ratio (aHR) for type 2 diabetes mellitus among cirrhotic individuals was 0.774 (CI: 0.715-0.8934). Alcoholic cirrhotic men had a significantly higher risk of type 2 diabetes (aHR 1.182, CI: 1.046-1.335) compared with non-cirrhotic individuals. Increased risks were seen in men (aHR 1.690; CI: 1.455-1.963) and women (aHR 1.715; CI: 1.113-2.645) with alcoholic cirrhosis compared to those with cirrhosis without alcohol.

**Conclusions:**

This study indicates that alcoholic cirrhosis is a significant risk factor for type 2 diabetes mellitus compared with cirrhosis without alcohol.

## INTRODUCTION

Diabetes mellitus is a global health issue. Alcoholic liver diseases have been associated with increased risk of type 2 diabetes [1]. The prevalence of diabetes in cirrhosis has been reported at 12.3-57% [2]. Previous publications have reported a high prevalence of liver diseases in diabetic patients and a high prevalence of diabetes in patients with liver disease [3, 4]. Increased risks of diabetes have also been reported in patients with cirrhosis due to hepatitis C (HCV) and alcoholic liver disease but not in patients with cirrhosis due to cholestatic liver disease [1, 4]. In Asia, more than half of the liver cirrhosis burden is linked to hepatitis B (HBV) and hepatitis C [5]. The prevalence of cirrhosis among Taiwanese patients with HBV is reported to be 49% [6]. Up to 30% of cirrhotic patients in Taiwan were seropositive for HBeAg while 73% had a serum HBV DNA level >10000 copies/ml [7]. A previous study including Chinese individuals in Taiwan reported that hepatitis B and C virus infection would act independently and synergistically in the development of liver cirrhosis [8]. Huang and colleagues reported that Taiwanese patients with chronic hepatitis B who develop diabetes are at increased risk of liver cirrhosis and its decompensation over time [9]. The exact incidence of liver cirrhosis especially in individual Asian countries is still unknown. From 2011-2013, the incidence rates of primary biliary cirrhosis in Korea were respectively 0.84, 0.92 and 0.87 per 100,000 population [10]. About 33,379 patients from 58 nationwide hospitals in Japan were diagnosed with liver cirrhosis in 2008 [11]. The prevalence rates of non-alcoholic liver disease (NAFLD) in South-Pacific Asia range from 12% to 24% in population subgroups and is about 11.4-41% in Taiwan [12]. About 69.4% of patients with NAFLD have been reported with type 2 diabetes mellitus (T2DM) [13]. In a prospective follow-up study including 8 663 men, heavy drinking was associated with a 2-fold increased risk of type 2 diabetes compared with moderate drinking [14]. It is worth mentioning that similar studies have not shown such relationships in women [15, 16].

Liver cirrhosis has been strongly associated with type 2 diabetes [3]. However, the association between T2DM and subcategories of cirrhosis is poorly understood. In this study, liver cirrhosis was categorized into two groups: alcoholic cirrhosis and cirrhosis without alcohol to investigate their association with diabetes mellitus.

## MATERIALS AND METHODS

### Data source

The data sources were the 2005 and 2010 Longitudinal Health Insurance Databases (LHID 2005 and 2010). These datasets contain de-identified secondary data which have been released to the public for research purposes. This study was exempted from full review by the Institutional Review Board. Informed consents were not applicable.

### Study population

**Figure 1:**
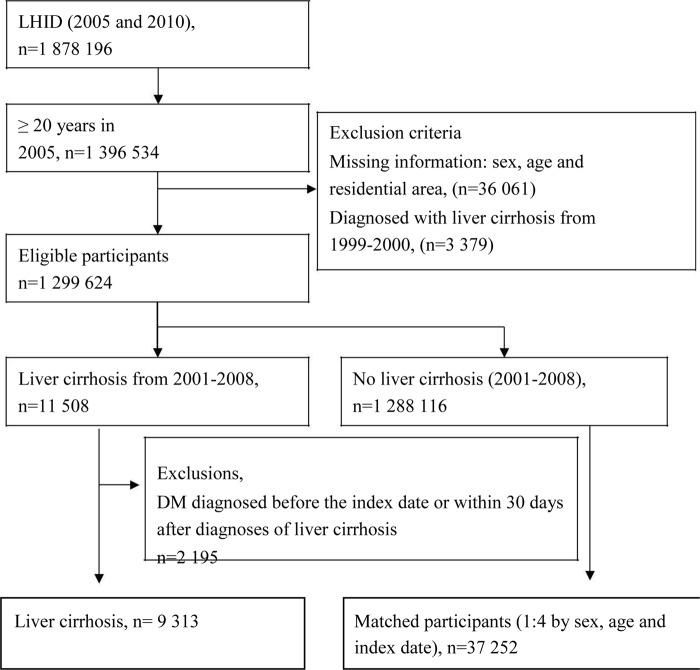
Flowchart of the study participants

The study participants included 1 878 196 enrollees sampled from the LHID 2005 and 2010 (Figure 1). All participants were aged 20 years and older. Newly diagnosed cirrhotic patients and their matched controls (comparison individuals) were identified from January 2001-December 2008 and matched 1:4 by age, sex and index date. The index date was the first date of cirrhosis detection. The event date was the first date of T2DM diagnosis or prescription of antihyperglycemic agents. Censoring occurred in case of death or withdrawal from the study. Patients were defined as having alcoholic cirrhosis if they had one inpatient and/or two outpatient claims with reported ICD-9 CM Code: 571.2. Cirrhosis without alcohol was identified using the ICD-9 CM Code: 571.5, 571.6. Patients were classified as having type 2 diabetes mellitus if they had one one inpatient and/or two outpatient claims with reported ICD-9 CM codes: 250.xx, 790.21, 790.22, 791.5x, A181. Patients were also classified as having T2DM if they were prescribed antihyperglycemic agents (alpha-glucosidase inhibitor, biguanides, insulin, meglitinides, sulfonylureas and thiazolidinediones) from 2001-2011.

### Baseline characteristics and statistical analysis

The demographic variables included sex, age, low-income, urbanization, and medications (antihypertensives such as ACE inhibitors, β-blocker and diuretics; antihyperlipidemics such as statins and fibrates as well as antiviral drugs). Co-morbidities included hepatitis B (ICD-9: 070.2, 070.3, V02.61), hepatitis C (ICD-9: 070.41, 070.44, 070.51, 070.54,070.7, V02.62), unspecified hepatitis (ICD-9: 070.9, 571.4, 571.8, 571.9), dyslipidaemia (ICD-9: 272), hypertension (ICD-9: 401-405), COPD (ICD-9: 490-505 506.4), cerebrovascular disease (ICD-9: 430-438), myocardial infarction (ICD-9: 410, 412) and renal disease (ICD-9: 582, 583-583.7 585, 586, 588).

The Chi-square test was used for the nominal variables between cirrhotic and comparison individuals. Pearson Chi-square was used to compare the differences in the distribution of variables between patients with alcoholic cirrhosis and those with cirrhosis without alcohol. A two-tailed t-test was used to compare the mean difference between individuals or groups. The incidence density (per 1 000 person months) of DM and its 95 % confidence interval (95% CI) were calculated. Cox proportional hazard model was used to estimate the hazard ratios (HR) and 95% CI of cirrhosis and other covariates. Because of the high rate of patients with cirrhosis, competing-risk regression was also performed using the Fine and Gray model. Statistical analyses were performed using SAS 9.3 software while a p-value <0.05 was considered as statistically significant.

## RESULTS

The final enrollment included 9 313 cirrhotic and 37 252 non-cirrhotic individuals (Figure 1). Table 1 shows the demographic characteristics of the study population. A significant proportion of cirrhotic patients were low-income earners (cirrhotic: 1.81% versus non-cirrhotic: 0.75%) and rural inhabitants (14.39 vs. 10.53%). Cirrhotic individuals also had a higher proportion of hepatitis B (22.33 vs. 2.12%), hepatitis C (16.41 vs. 0.80%), unspecified hepatitis (59.74 vs. 12.76%), dyslipidaemia (16.65 vs. 12.45%), hypertension (33.36 vs. 25.94%), COPD (23.70% vs. 17.97%), cerebrovascular disease (10.32 vs. 7.50%), myocardial infarction (1.12 vs. 0.86%), renal disease (7.61 vs. 3.37%), antihypertensive medications, and antihyperlipidemic agents. The proportion of men with alcoholic cirrhosis was higher than that of those with *cirrhosis without alcohol* (92.97% vs. 63.34%).

**Table 1:** Basic characteristics of the study participants

	Control individuals	Cirrhotic individuals		
	No cirrhosis (*n* = 37 252)	All^⌧^ (9 313)	Nonalcoholic *n* = 7 064	Alcoholic *n* = 2 249
Sex, *n* (%)^#^				
Male	26 260 (70.49)	6 565 (70.49)	4 474 (63.34)	2 091 (92.97)
Female	10 992 (29.51)	2 748 (29.51)	2 590 (36.66)	158 (7.03)
Age in 2005 (Mean±SD)	54.49±14.92	54.49±14.92	56.87±15.06	47±11.65
Low-income, *n* (%)^*,#^				
No	36 972 (99.25)	9 144 (98.19)	6 975 (98.74)	2 169 (96.44)
Yes	280 (0.75)	169 (1.81)	89 (1.26)	80 (3.56)
Residents, *n* (%)^*,#^				
Urban	21 729 (58.33)	4 913 (52.75)	3 823 (54.12)	1 090 (48.47)
Sub-urban	11 601 (31.14)	3 060 (32.86)	2 285 (32.35)	775 (34.46)
Rural	3 922 (10.53)	1 340 (14.39)	956 (13.53)	384 (17.07)
Comorbidity, *n* (%)				
Hepatitis B^*,#^	790 (2.12)	2 080 (22.33)	1 750 (24.77)	330 (14.67)
Hepatitis C^*,#^	298 (0.80)	1 528 (16.41)	1 356 (19.2)	172 (7.65)
Unspecified hepatitis^*,#^	4 753 (12.76)	5 564 (59.74)	4 325 (61.23)	1 239 (55.09)
Dyslipidemia^*^	4 638 (12.45)	1 551 (16.65)	1 192 (16.87)	359 (15.96)
Hypertension^*,#^	9 662 (25.94)	3 107 (33.36)	2 580 (36.52)	527 (23.43)
COPD^*,#^	6 695 (17.97)	2 207 (23.7)	1 846 (26.13)	361 (16.05)
Cerebrovascular disease^*,#^	2 795 (7.50)	961 (10.32)	823 (11.65)	138 (6.14)
Myocardial infarction^*^	322 (0.86)	104 (1.12)	85 (1.2)	19 (0.84)
Renal disease^*,#^	1 257 (3.37)	709 (7.61)	627 (8.88)	82 (3.65)

^⌧^Included alcoholic cirrhosis and cirrhosis without alcohol

*Significantly different between cirrhotic and comparison patients, *p* < 0.05

#Significantly different between patients with alcoholic cirrhosis and cirrhosis without alcohol *p* < 0.05

The proportion of participants with alcoholic cirrhosis and cirrhosis without alcohol were as follows; low income (3.56 vs. 1.26%), rural inhabitants (17.07 vs. 13.53%), hepatitis B (14.67 vs. 24.77%), hepatitis C (7.65 vs. 19.20%), unspecified hepatitis (55.09 vs. 61.23%), hypertension (23.43 vs. 36.52%), COPD (16.05 vs. 26.13%), cerebrovascular disease (6.14 vs. 11.65%), and renal disease (8.88 vs. 3.65%). In general, 2 315 cirrhotic and 7 289 non-cirrhotic individuals were diagnosed with T2DM from 2001-2010. Among the cirrhotic patients who were newly diagnosed with DM, 1 562 had alcoholic cirrhosis while 753 had cirrhosis without alcohol. The incidence density (per 1 000 person months) of T2DM was 1.88 (1.76-2.01) for individuals with cirrhosis, 1.62 (1.48-1.78) for those with cirrhosis without alcohol, 2.92 (CI 2.64-3.23) for those with alcoholic cirrhosis, and 1.14 (CI: 1.09-1.20) for the comparison individuals (2001-2011). The adjusted HR for T2DM among cirrhotic patients was 0.774 (CI 0.715-0.838) compared to non-cirrhotic individuals (Table 2). In addition, unspecified viral hepatitis was found to be a significant risk factor for T2DM (aHR 1.233, CI 1.147-1.326). However, the hazard ratio was 0.807 (CI 0.711-0.915) in patients with HBV. Compared with the non-cirrhotic individuals, the hazard ratios for T2DM were 1.182 (CI 1.046-1.335) in men and 0.889 (CI 0.601-1.345) in women with alcoholic cirrhosis (Table 3). Those of men and women with cirrhosis without alcohol were 0.723 (CI 0.645-0.810) and 0.549 (CI 0.469-0.644), respectively. Table 4 shows the adjusted hazard ratios for T2DM in patients with alcoholic cirrhosis and cirrhosis without alcohol as the reference group. The hazards ratios were 1.929 (CI: 1.742-2.136) and 1.787 (CI 1.339-2.383) for the alcoholic cirrhotic men and women, respectively.

**Table 2:** Hazards ratios and 95% confidence interval of type 2 diabetes among patients with liver cirrhosis

	aHR* (95% CI)	*p*-value
Cirrhosis (Reference: Comparison group)		
Cirrhotic individuals	0.774(0.715-0.838)	<.0001
Low-income (Reference: No)		
Yes	1.432(1.138-1.803)	0.0022
Residents (Reference: Urban)		
Sub-urban	0.994(0.913-1.082)	0.8934
Rural	0.958(0.904-1.015)	0.1442
Comorbidity (Reference: No)		
Hepatitis B	0.807(0.711-0.915)	0.0008
Hepatitis C	0.904(0.771-1.060)	0.2121
Unspecified hepatitis	1.233(1.147-1.326)	<.0001
Dyslipidemia	1.650(1.519-1.792)	<.0001
Hypertension	1.732(1.602-1.873)	<.0001
COPD	1.111(1.036-1.192)	0.0033
Cerebrovascular disease	0.958(0.851-1.078)	0.4764
Myocardial infarction	1.203(0.855-1.691)	0.2886
Renal disease	1.176(1.020-1.357)	0.0257

Abbreviation: CI, confidence interval, COPD, chronic obstructive pulmonary disease.

* aHR represented adjusted hazard ratios: adjusted for low-income, residents, comorbidity, antihypertensive, antihyperlipidemics and antiviral drugs

**Table 3:** Hazard ratios and 95% confidence intervals of type 2 diabetes mellitus associated with cirrhosis and covariates in men and women

	Male		Female		
	aHR* (95% CI)	*p*-value	aHR* (95% CI)	*p*-value	
Cirrhosis (Reference: No cirrhosis)					
Cirrhosis without alcohol	0.723(0.645-0.810)	<.0001	0.549(0.469-0.644)		<.0001
Alcoholic	1.182(1.046-1.335)	0.0072	0.899(0.601-1.345)		0.6042
Low income (Reference: No)					
Yes	1.285(0.992-1.666)	0.0577	1.582(0.954-2.625)		0.0756
Residents (Reference: Urban)					
Sub-urban	0.949(0.885-1.017)	0.1402	0.973(0.876-1.079)		0.6022
Rural	1.004(0.907-1.112)	0.9355	0.935(0.803-1.088)		0.3868
Comorbidity (Reference: No)					
Hepatitis B	0.793(0.684-0.919)	0.0020	0.979(0.763-1.255)		0.8653
Hepatitis C	1.022(0.833-1.254)	0.8323	0.904(0.699-1.168)		0.4394
Unspecified hepatitis	1.215(1.114-1.325)	<.0001	1.278(1.121-1.457)		0.0002
Dyslipidemia	1.691(1.523-1.877)	<.0001	1.531(1.336-1.755)		<.0001
Hypertension	1.708(1.547-1.884)	<.0001	1.753(1.541-1.993)		<.0001
COPD	1.092(1.001-1.192)	0.047	1.170(1.039-1.317)		0.0094
Cerebrovascular disease	0.946(0.813-1.101)	0.4745	1.002(0.830-1.209)		0.9838
Myocardial infarction	0.896(0.566-1.419)	0.6402	2.250(1.367-3.704)		0.0014
Renal disease	1.227(1.027-1.465)	0.0241	1.163(0.914-1.481)		0.2195

Abbreviation: CI, confidence interval, COPD, chronic obstructive pulmonary disease.

* aHR represented adjusted hazard ratios: adjusted for low-income, residents, comorbidity, antihypertensive, antihyperlipidemics and antiviral drugs

**Table 4:** Adjusted hazard ratios of type 2 diabetes mellitus in patients with alcoholic cirrhosis and in patients with cirrhosis without alcohol as the reference group

	Male		Female		
	aHR* (95% CI)	*p*-value	aHR* (95% CI)	*p*-value	
Cirrhosis (Reference: cirrhosis without alcohol)					
Alcoholic	1.690(1.455-1.963)	<.0001	1.715(1.113-2.645)		0.0146
Low income (Reference: No)					
Yes	1.157(0.763-1.754)	0.4918	1.643(0.544-4.968)		0.3788
Residents (ref: Urban)					
Sub-urban	0.916(0.782-1.073)	0.2794	0.866(0.665-1.129)		0.2888
Rural	0.953(0.773-1.176)	0.6561	0.839(0.579-1.216)		0.3543
Comorbidity (Reference: No)					
Hepatitis B	0.877(0.729-1.054)	0.1620	0.940(0.671-1.316)		0.7177
Hepatitis C	1.048(0.824-1.333)	0.7043	1.010(0.741-1.377)		0.9501
Unspecified hepatitis	1.148(0.989-1.332)	0.0698	1.217(0.933-1.588)		0.1483
Dyslipidemia	1.312(1.077-1.597)	0.0069	1.632(1.200-2.221)		0.0018
Hypertension	1.735(1.429-2.106)	<.0001	1.571(1.160-2.127)		0.0035
COPD	0.992(0.823-1.196)	0.9301	1.144(0.861-1.519)		0.3538
Cerebrovascular disease	0.967(0.717-1.302)	0.823	0.882(0.559-1.393)		0.5914
Myocardial infarction	0.626(0.235-1.663)	0.3471	2.325(0.721-7.502)		0.1579
Renal disease	1.144(0.819-1.597)	0.4312	1.266(0.818-1.961)		0.2904

Abbreviation: CI, confidence interval, COPD, chronic obstructive pulmonary disease.

* aHR represented adjusted hazard ratios: adjusted for low-income, residents, comorbidity, antihypertensive, antihyperlipidemics and antiviral drugs

## DISCUSSION

In this large nationwide cohort study, a positive association was found between alcoholic cirrhosis as a risk factor for the development of type 2 diabetes. Previous studies have reported significant proportions of cirrhotic patients with diabetes [17–21]. Nonetheless, cirrhosis was not analyzed by subgroups. In a case-control study, 47% of patients with cryptogenic cirrhosis had T2DM compared to 22% of the controls [22]. Another study showed that the risks of T2DM were 7.6 and 5.3 times higher in hepatitis C and alcoholic cirrhosis, respectively [1]. However, only a few variables were considered during analysis.

In this study, when categorized by sex, a higher risk was found particularly in men with alcoholic cirrhosis. However, after including cirrhosis subgroups in the competing risk analysis, T2DM risk was found to be significantly higher in both men and women. Heavy amounts of alcohol have shown direct diabetogenic effects [23]. One study has reported a significant association between alcohol intake and T2DM with a relative risk of 1.5 per 137.8 g of alcohol intake in the past week [15]. Other studies found that moderate alcohol consumption was negatively correlated with T2DM even with an intake beyond 48 g / day [24]. Alcohol consumption has been inversely associated with fasting and post-load insulin levels [25, 26]. It has also been reported that excess alcohol may reduce insulin-mediated glucose uptake [21] and can cause injury to pancreatic islet β-cells resulting in type 2 diabetes mellitus [27]. In addition, alcohol can also cause fatty liver disease leading to dysfunction of the mitochondria, thereby increasing the risk of T2DM [28, 29].

Participants who used higher doses of antihypertensive and antihyperlipidemic agents were also found with higher risks of T2DM when compared with non-users. Past studies have associated diuretics and β-blocker with increased risk of diabetes [26]. When analyzed by sex, we found that long-term use of ACEI, β-blocker, diuretic and fibrates women were positively associated with T2DM in both men and women. Therefore, it was important that such confounders be adjusted.

We also found a lower risk of T2DM among patients suffering from HBV. The HR was lower but not significant for HCV. However, unspecified hepatitis was significantly associated with increased risk of T2DM. Previous studies have reported diverging conclusions on the association between HCV and T2DM [30]. Results from a meta-analysis indicate that HBV itself may not be pro-diabetic [31]. Among the cirrhotic individuals in this study, about 22.33 % had HBV. However, whether all the cases of cirrhosis were HBV-derived cannot be fully explained.

Hepatogenous diabetes is a common complication of liver cirrhosis [32, 33]. As noted earlier, recent studies have reported increased risk of diabetes in patients with liver cirrhosis due to hepatitis C and alcoholic liver disease. However, no increased risk was found in patients with liver cirrhosis due to cholestatic liver disease [1]. Nonalcoholic fatty liver disease [34], chronic viral hepatitis [35, 36], hemochromatosis [37], alcoholic liver disease [1, 15] and cirrhosis [27] have been associated with T2DM. However, their relationships may be independent of the lifestyle risk factors and other metabolic diseases [15].

Our study made use of a large sample size with a longer period of follow-up. However, it was not without limitations. First, the severity of cirrhosis could not be determined. Second, there was a dearth of information about lipid and blood glucose levels. Lastly, information on alcohol intake was not available. Alcoholic cirrhosis was defined based on the ICD-9 CM codes, and hence information bias cannot be ruled out.

## CONCLUSIONS

In summary, patients with alcoholic cirrhosis were found with a higher risk of type 2 diabetes mellitus compared to those with cirrhosis without alcohol. Monitoring of blood glucose levels is recommended for patients with cirrhosis.

## References

[1] Zein NN, Abdulkarim AS, Wiesner RH, Egan KS, Persing DH (2000). Prevalence of diabetes mellitus in patients with end-stage liver cirrhosis due to hepatitis C, alcohol, or cholestatic disease. J Hepatol.

[2] Trombetta M, Spiazzi G, Zoppini G, Muggeo M (2005). Review article: type 2 diabetes and chronic liver disease in the Verona diabetes study. Aliment Pharmacol Ther.

[3] Tolman KG, Fonseca V, Dalpiaz A, Tan MH (2007). Spectrum of liver disease in type 2 diabetes and management of patients with diabetes and liver disease. Diabetes Care.

[4] Hsieh PS, Hsieh YJ (2011). Impact of liver diseases on the development of type 2 diabetes mellitus. World J Gastroenterol.

[5] Mokdad AA, Lopez AD, Shahraz S, Lozano R, Mokdad AH, Stanaway J, Murray CJ, Naghavi M (2014). Liver cirrhosis mortality in 187 countries between 1980 and 2010: a systematic analysis. BMC Med.

[6] Yang SS (2016). Alcoholic Liver Disease in the Asian–Pacific Region with High Prevalence of Chronic Viral Hepatitis. J Med Ultrasound.

[7] Chen YC, Chu CM, Yeh CT, Liaw YF (2007). Natural course following the onset of cirrhosis in patients with chronic hepatitis B: a long-term follow-up study. Hepatol Int.

[8] Tsai JF, Chang WY, Jeng JE, Ho MS, Wang LY, Hsieh MY, Chen SC, Chuang WL, Lin ZY, Tsai JH (1993). Hepatitis C virus infection as a risk factor for non-alcoholic liver cirrhosis in Taiwan. J Med Virol.

[9] Huang YW, Wang TC, Lin SC, Chang HY, Chen DS, Hu JT, Yang SS, Kao JH (2013). Increased risk of cirrhosis and its decompensation in chronic hepatitis B patients with newly diagnosed diabetes: a nationwide cohort study. Clin Infect Dis.

[10] Kim KA, Choi H, Ki M, Jeong SH (2015). P1171: Epidemiology and disease burden of primary biliary cirrhosis in South Korea: A nationwide, population-based study. J Hepatol.

[11] Michitaka K, Nishiguchi S, Aoyagi Y, Hiasa Y, Tokumoto Y, Onji M, Japan Etiology of Liver Cirrhosis Study Group (2010). Etiology of liver cirrhosis in Japan: a nationwide survey. J Gastroenterol.

[12] Hsu CS, Kao JH (2012). Non-alcoholic fatty liver disease: an emerging liver disease in Taiwan. J Formos Med Assoc.

[13] Leite NC, Salles GF, Araujo AL, Villela-Nogueira CA, Cardoso CR (2009). Prevalence and associated factors of non-alcoholic fatty liver disease in patients with type-2 diabetes mellitus. Liver Int.

[14] Wei M, Gibbons LW, Mitchell TL, Kampert JB, Blair SN (2000). Alcohol intake and incidence of type 2 diabetes in men. Diabetes Care.

[15] Holbrook TL, Barrett-Connor E, Wingard DL (1990). A prospective population-based study of alcohol use and non-insulin-dependent diabetes mellitus. Am J Epidemiol.

[16] Stampfer MJ, Colditz GA, Willett WC, Manson JE, Arky RA, Hennekens CH, Speizer FE (1988). A prospective study of moderate alcohol drinking and risk of diabetes in women. Am J Epidemiol.

[17] Kuriyama S, Miwa Y, Fukushima H, Nakamura H, Toda K, Shiraki M, Nagaki M, Yamamoto M, Tomita E, Moriwaki H (2007). Prevalence of diabetes and incidence of angiopathy in patients with chronic viral liver disease. J Clin Biochem Nutr.

[18] Megyesi C, Samols E, Marks V (1967). Glucose tolerance and diabetes in chronic liver disease. Lancet.

[19] Kingston ME, Ali MA, Atiyeh M, Donnelly RJ (1984). Diabetes mellitus in chronic active hepatitis and cirrhosis. Gastroenterology.

[20] Chen YW, Chen HH, Wang TE, Chang CW, Chang CW, Chen WC, Wu CJ (2011). The dissociation between the diabetes and both Child-Pugh score and in-hospital mortality in cirrhotic patients due to hepatitis B, hepatitis C, or alcoholic. Hepatol Int.

[21] Garcia-Compean D, Jaquez-Quintana JO, Gonzalez-Gonzalez JA, Maldonado-Garza H (2009). Liver cirrhosis and diabetes: risk factors, pathophysiology, clinical implications and management. World J Gastroenterol.

[22] Poonawala A, Nair SP, Thuluvath PJ (2000). Prevalence of obesity and diabetes in patients with cryptogenic cirrhosis: a case-control study. Hepatology.

[23] Howard AA, Arnsten JH, Gourevitch MN (2004). Effect of alcohol consumption on diabetes mellitus: a systematic review. Ann Intern Med.

[24] Koppes LL, Dekker JM, Hendriks HF, Bouter LM, Heine RJ (2005). Moderate alcohol consumption lowers the risk of type 2 diabetes: a meta-analysis of prospective observational studies. Diabetes Care.

[25] Mayer EJ, Newman B, Quesenberry CP, Friedman GD, Selby JV (1993). Alcohol consumption and insulin concentrations. Role of insulin in associations of alcohol intake with high-density lipoprotein cholesterol and triglycerides. Circulation.

[26] Elliott WJ, Meyer PM (2007). Incident diabetes in clinical trials of antihypertensive drugs: a network meta-analysis. Lancet.

[27] Hickman IJ, Macdonald GA (2007). Impact of diabetes on the severity of liver disease. Am J Med.

[28] Mantena SK, King AL, Andringa KK, Eccleston HB, Bailey SM (2008). Mitochondrial dysfunction and oxidative stress in the pathogenesis of alcohol- and obesity-induced fatty liver diseases. Free Radic Biol Med.

[29] Lowell BB, Shulman GI (2005). Mitochondrial dysfunction and type 2 diabetes. Science.

[30] Gastaldi G, Goossens N, Clément S, Negro F (2016). Current level of evidence on causal association between Hepatitis C virus and type 2 diabetes: A review. J Adv Res.

[31] Zhang J, Shen Y, Cai H, Liu YM, Qin G (2015). Hepatitis B virus infection status and risk of type 2 diabetes mellitus: A meta-analysis. Hepatol Res.

[32] Holstein A, Hinze S, Thiessen E, Plaschke A, Egberts EH (2002). Clinical implications of hepatogenous diabetes in liver cirrhosis. J Gastroenterol Hepatol.

[33] Picardi A, D’Avola D, Gentilucci UV, Galati G, Fiori E, Spataro S, Afeltra A (2006). Diabetes in chronic liver disease: from old concepts to new evidence. Diabetes Metab Res Rev.

[34] Younossi ZM, Gramlich T, Matteoni CA, Boparai N, McCullough AJ (2004). Nonalcoholic fatty liver disease in patients with type 2 diabetes. Clin Gastroenterol Hepatol.

[35] Caronia S, Taylor K, Pagliaro L, Carr C, Palazzo U, Petrik J, O’Rahilly S, Shore S, Tom BD, Alexander GJ (1999). Further evidence for an association between non-insulin-dependent diabetes mellitus and chronic hepatitis C virus infection. Hepatology.

[36] White DL, Ratziu V, El-Serag HB (2008). Hepatitis C infection and risk of diabetes: a systematic review and meta-analysis. J Hepatol.

[37] Conte D, Manachino D, Colli A, Guala A, Aimo G, Andreoletti M, Corsetti M, Fraquelli M (1998). Prevalence of genetic hemochromatosis in a cohort of Italian patients with diabetes mellitus. Ann Intern Med.

